# Carbon Gels-Modified TiO_2_: Promising Materials for Photocatalysis Applications

**DOI:** 10.3390/ma13071734

**Published:** 2020-04-08

**Authors:** Dongge Ma, Jundan Li, Anan Liu, Chuncheng Chen

**Affiliations:** 1 School of Science, Beijing Technology and Business University, Beijing 100048, China; 1930102009@st.btbu.edu.cn; 2 Basic Experimental Center for Natural Science, University of Science and Technology Beijing, Beijing 100083, China; liuanan@ustb.edu.cn; 3 Key Laboratory of Photochemistry, Beijing National Laboratory for Molecular Sciences, Institute of Chemistry, Chinese Academy of Sciences, Beijing 100190, China; ccchen@iccas.ac.cn

**Keywords:** carbon gels, photocatalysis, photo-induced carrier separation, pollutants degradation

## Abstract

Carbon gels are a kind of porous organic polymer, which play pivotal roles in electrode, supercapacitor, hydrogen storage, and catalysis. Carbon gels are commonly prepared by the condensation of resorcinol and formaldehyde. The as-prepared polymers are further aged and sintered at a high temperature in an inert atmosphere to form cross-linked and intertwined porous structures. Owing to its large specific area and narrow pore size distribution, this kind of material is very appropriate for mass transfer, substrate absorption, and product desorption from the pores. In recent years, carbon gels have been discovered to function as effective hybrid materials with TiO_2_ for photocatalytic applications. They could act as efficient deep-traps for photo-induced holes, which decreases the recombination probability of photo-induced carriers and lengthens their lifetime. In this mini-review, we will discuss the state-of-the-art paragon examples of carbon gels/TiO_2_ composite materials applied in photo(electro)catalysis. The major challenges and gaps of its application in this field will also be emphasized.

## 1. Introduction

Nowadays, environmental pollution and energy shortage are two main crises facing the whole world. The excessive utilization of fossil fuels produces various pollutants, which was discharged into water and air environments. There is an urgent need for the use of green and renewable energy resources for human society. Sunlight, considered as an inexhaustible and environmentally friendly energy source, has aroused the attention from both academics and industries. As Fujishima and Honda first reported TiO_2_ could be used to catalyze the water-splitting process evolving hydrogen and oxygen under sunlight illumination in 1972 [1], and Carey et al. initially used TiO_2_ photocatalysis as an advanced oxidation technique for the removal of pollutants in aqueous solution [2,3], photocatalysis has been developed rapidly and becomes the focus of various disciplines including catalysis [4,5,6,7], materials chemistry [8,9,10], environmental chemistry [11], energy chemistry [12,13], surface chemistry [14,15,16], and processing chemistry and chemical engineering [17,18]. Among various photocatalysts, TiO_2_ being extremely stable under light irradiation, highly acidic, and basic conditions; non-toxic, earth-abundant, and easily-recyclable; and reusable without much loss of activity, has been profoundly investigated from the 1970s up till now [19,20,21,22,23,24,25,26,27]. Moreover, with the high photo-induced hole oxidation potential (E_vb_^+^ = 2.7 V vs. NHE (Normal Hydrogen Electrode) at pH = 7); the ability to produce similarly highly oxidative OH• radicals (E = 2.8 V vs. NHE), H_2_O_2_, and HOO•; and the appropriate photo-induced conduction band electron reduction potential (E = −0.5 V vs. NHE at pH = 7), which could be easily trapped by dioxygen generating superoxide radical anion, all of these reactive oxygen species, along with the photo-induced holes on TiO_2_ surface, could almost decompose all the organic pollutants by unselective thorough mineralization with sequential hydrogen abstraction; halogen abstraction; and addition to R•, C=C, and C≡C bonds until all the organic pollutants finally transform to CO_2_, H_2_O, and inorganic ion species [7,11,28]. Although TiO_2_ photocatalysis has garnered a plethora of successful examples in water environment remediation applications [29,30,31,32,33], there are still gaps and shortcomings that need to be overcome for TiO_2_ photocatalysis to be applied in real industrial processes. The following main issues need to be addressed. Firstly, owing to the wide band gap (E_g_ = 3.2 eV for anatase and 3.0 eV for rutile), TiO_2_-based nanomaterial could only be excited under UV (ultra-violet) irradiation (λ < 387.5 nm). This means that only less than 5% of the solar spectrum energy could be utilized by TiO_2_ photocatalysis. Approximately 95% of the solar energy is wasted as heat. Furthermore, current TiO_2_ photocatalysis displays a low quantum yield, even in UV spectrum. Poor photon energy to chemical energy efficiency is obtained for the existing TiO_2_ system. The quantum yield is in the range below 20%. The poor photocatalysis performance of TiO_2_ is attributed to the following factors: (1) poor visible-light absorption, (2) facile hole-electron recombination, and (3) poor adsorption and catalytic activity towards non-polar and hydrophobic compounds. To overcome these obstacles, various strategies have been developed. Metal and non-metal atoms [34] doping such as Fe [35], Cu [36], Ni [37] and N [38,39,40], C [41], and S [42], which could introduce extra traps below the bottom of the conduction band and the top of the valence band, were incorporated in lowering the band gap to facilitate visible-light absorption ability [43]. However, the addition of these dopant atoms could in another way decrease its photocatalytic efficiency, as the trapping sites would also be the recombination sites for electrons and holes. Coupling other photocatalysts with TiO_2_ to construct heterojunction structure is another approach to improve TiO_2_ photocatalysis performance. A number of inorganic and organic semiconductor photocatalysts, including CdS [44], BiVO_4_ [45], AgBr [46], RGO [47], and g-C_3_N_4_ [48], have been combined with TiO_2_ to generate a hybrid photocatalyst system, which displays excellent performance for various photocatalytic applications including water-splitting, CO_2_ reduction, water and air decontamination, and organic synthesis [49,50,51,52].

**Figure 1:**
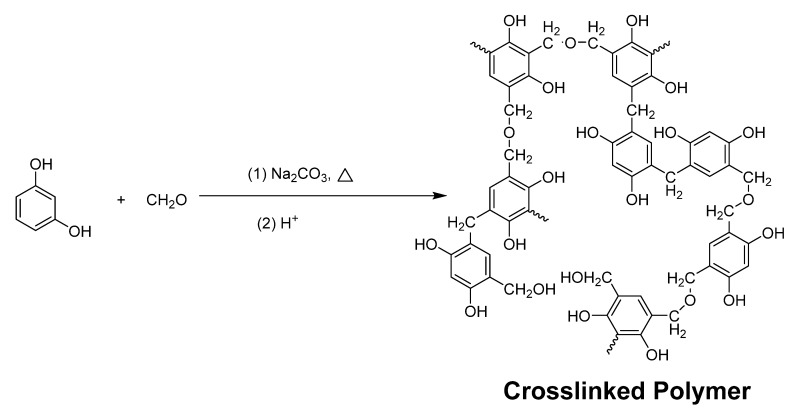
A schematic diagram of the reaction of resorcinol and formaldehyde to synthesize carbon aerogel. Copied with permission from [54], Copyrights 1989 Springer [54].

Carbon gels, as a kind of aerogels, were discovered as early as 1931 by Kistler [53]. The author successfully prepared the gels using the supercritical liquid evaporation method, which guaranteed that the jelly internal structure did not experience either change or shrinkage. This is a milestone discovery for colloid chemistry. However, the synthesis and preparation of carbon gels was not rediscovered and researched until more than half a century later. Pekala and co-workers innovatively developed the four-step procedure to synthesize resorcinol–formaldehyde (RF) gels as shown in Figure 1 [54]. Firstly, resorcinol and formaldehyde were polycondensed in an alkaline solution. Then, the as-formed RF mixtures were subjected to aging conditions at 85 °C for several days. Later, the acid-treatment and solvent-exchange processes were required before the key critical-point drying procedure. Finally, CO_2_ was introduced to flow in and replace the residue solvent molecules at 45 °C. The dried RF gels were characterized by various techniques to prove the existence of the organic aerogel structure. This example resembles the renaissance of organic carbon gels [55,56,57,58,59]. Nowadays, carbon gels have been extensively investigated and applied in hydrogen and methane storage [60,61,62,63,64,65,66,67,68,69], electric energy storage [70,71,72,73], thermal transport [74,75], and catalysis [76,77,78]. Carbon gels have been demonstrated as ideal materials for these applications owing to their large specific surface area, high electric conductivity, and porous structure [79,80]. These features would also be critical merits for catalyst and co-catalyst materials for photocatalysis applications [81,82]. Carbon gels have been successfully incorporated into TiO_2_ photocatalysis to improve the overall performance for photocatalytic water-splitting and environment remediation [83]. This review will discuss the state-of-the-art paragon examples of carbon gels-modified TiO_2_-nanomaterials including the preparation, characterization, and activity of these composite photocatalysts. Furthermore, the gaps and challenges of this area will also be outlined.

## 2. Carbon Gels-Modified TiO_2_ Photocatalysis

Owing to their large specific surface area, ordered porous structure, and high electric conductivity, carbon gels have been applied in various fields including hydrogen storage, electrode materials, and heterogeneous catalyst support. Although carbon gels are often used as conductive materials, they also can be used to construct a composite semiconductor photocatalyst with TiO_2_.

**Figure 2:**
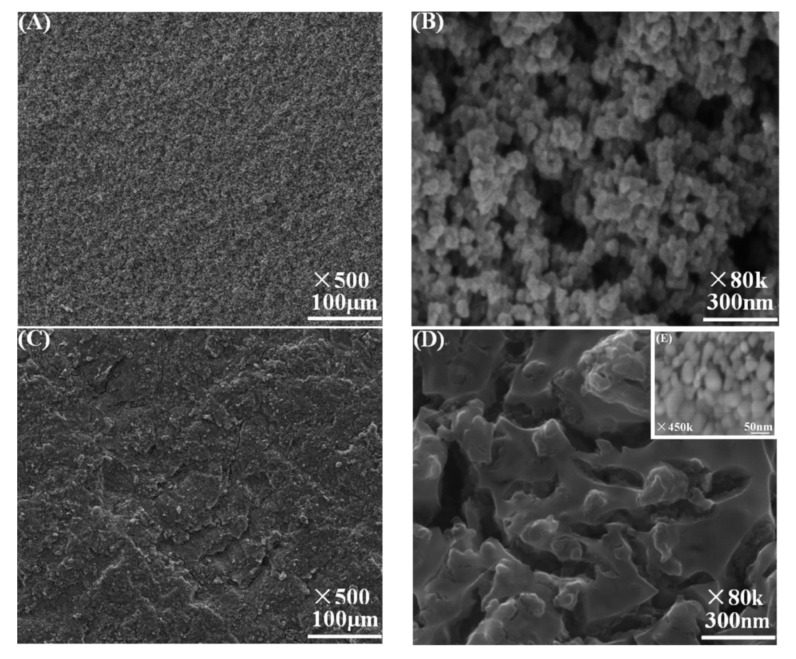
Scanning electronic microscopy (SEM) images of carbon aerogel (CA) (**A** × 500, **B** × 80 k) and TiO_2_/CA (**C** × 500, **D** × 80 k, **E** × 450 k). Copied with the permission from [84], Copyrights 2010 ACS [84].

In 2010, Zhao and co-workers firstly used carbon gels to modify the TiO_2_ photocatalyst to improve its adsorption and conductivity performance [84]. They prepared a TiO_2_/carbon aerogel (CA) photoelectrode material. The CA was synthesized by the base-catalyzed polycondensation between resorcinol and formaldehyde. The as-synthesized wet gels were initially solvent-exchanged by acetone to replace water. The organic gel was transformed to CA by heating in an argon atmosphere at 950 °C. The CA material was immersed into the sol–gel process of TiO_2_ formation. The as-formed mixed TiO_2_/CA was sintered at different high temperatures to yield the final photoelectrode material. Various characterization techniques such as scanning electronic microscopy (SEM), X-ray diffraction (XRD), Raman spectrometry, and N_2_ isothermal adsorption and desorption experiments were applied to determine the morphology (as shown in Figure 2), crystal structure, and porosity property. The TiO_2_/CA photoelectrode possesses both excellent photocatalytic properties owing to the anatase TiO_2_ semiconductor photoresponsive component and outstanding electrochemical properties of CA material, such as its extremely high conductivity, low electrochemical impedance, and large specific surface area. Thus, the TiO_2_/CA photoelectrode demonstrated much higher photocurrent density and degradation efficiency for highly opaque methylene blue wastewater under a −0.6 V potential bias and 365 nm light irradiation compared with the TiO_2_/ITO photoelectrode. The authors attributed the high efficiency to the following factors. Firstly, applying −0.6 V bias would form an electrical double layer between the electrode and bulk solution, causing the pollutants methylene blue, which possesses positive charges, to adsorb on TiO_2_/CA electrode surface. The excellent conductivity of CA materials would enhance the electrosorption process and the large specific surface area would promote the dispersion of TiO_2_ nano-crystallite on the electrode film. The electrosorption effect would increase the transparency of the opaque wastewater, which would greatly increase the photocatalytic degradation efficiency of TiO_2_.

**Figure 3:**
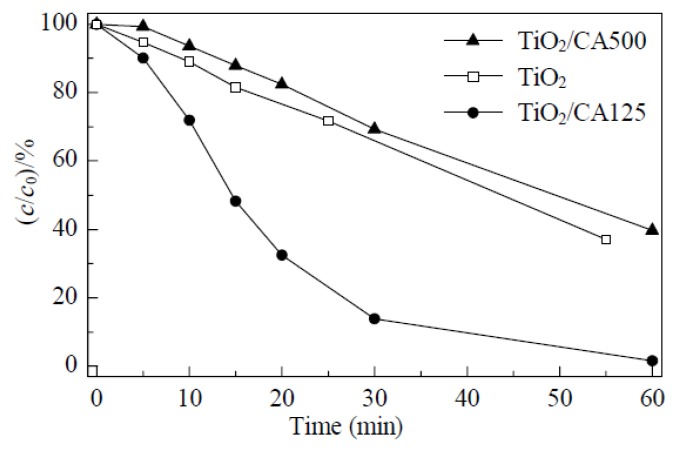
The photocatalytic degradation curves of methyl orange dye over TiO_2_ and TiO_2_/CA photocatalysts. Copied with the permission from [85], Copyrights 2011 Elsevier [85].

In 2011, Wu and co-workers reported that mesoporous and macroporous carbon aerogels could couple with TiO_2_ to construct a hybrid CA/TiO_2_ composite photocatalyst [85]. The composite photocatalyst was prepared by a sol–gel process mixing Ti(iPrO)_4_ with CA in an alcoholic solution. The as-prepared CA was sintered at 400 °C under N_2_ atmosphere. Two CA materials with different pore diameters were hybridized with TiO_2_. The one with mesoporous structure displayed much higher activity on methyl orange degradation compared with pristine TiO_2_ and TiO_2_/CA with macroporous structure as shown in Figure 3. By analyzing XRD (X-ray diffraction), SEM (scanning electron microscopy), and BET (Brunner-Emmet-Teller measurements) experimental results, the authors determined that the mesoporous structure of TiO_2_/CA125 photocatalyst promoted the substrates adsorption and products desorption because of the suitable pore size. The micropores could not accommodate the considerably large methyl orange dye molecule entering the pore, while the macropore is too large to confine the dye molecule inside the pore for effective adsorption and surface photocatalytic reaction.

**Figure 4:**
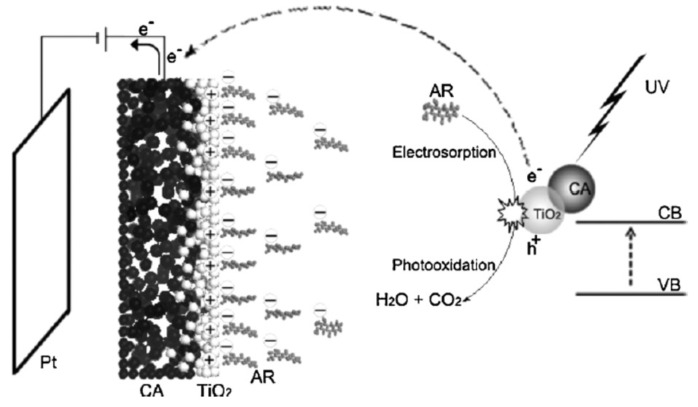
Mechanism of photocatalysis enhanced electroadsorption (PES) degradation of alizarin red (AR) dye on the TiO_2_/CA photoelectrode. Copied with the permission from [86], Copyrights 2011 Elsevier [86]. CB: Conduction Band. VB: Valence band.

In 2011, Zhao and co-workers reported that CA/TiO_2_ hybrid material could act as a very promising candidate for a photocatalysis enhanced electroadsorption (PES)-based dye pollutant elimination process [86]. They prepared the CA/TiO_2_ electrode by dispersing the as-prepared CA material into titania tetrabutylate sol–gel. The CA material was synthesized from the poly-condensation of resorcinol and formaldehyde, the displacement of water by immersing in acetone, and final sintering under argon at a high temperature forming the final porous aerosol microstructure. The morphology, surface composition, and properties were characterized by SEM, EDS (Energy Dispersive Spectroscopy), TG/DTA (Thermogravimetric Analysis/Differential Thermal Analysis), BET, EIS (Electrochemical Impedance Spectroscopy), and Raman spectrometry. Furthermore, the material demonstrated excellent alizarin red (AR) dye pollutant elimination ability by combining adsorption, electroadsorption, and photocatalysis in a three-in-all strategy. In a 400 mg/L high concentration AR pollutant solution, the hybrid material exhibited 97.3% TOC (Total Organic Carbon) removal in 240 min for the PES process in comparison with 59% in the photocatalysis process without applied electric field and 22% in the electrosorption process without illumination. Not only did it possess high AR removal efficiency, but this material also exhibited very good stability, providing 92.3% AR removal after five cycles. Moreover, the authors proposed a mechanism for the synergistic effect of photocatalysis and electrosorption as shown in Figure 4. The adsorbed dye molecules labile bonds were initially cleaved via single-electron-transfer by TiO_2_ photo-induced hole species or hydrogen abstraction by hydroxyl radicals and other reactive oxygen species (ROS), and the dye molecule radical cations were hydroxylated and mineralized step by step via radical addition, atom abstraction, and bond cleavage mechanism. The photo-induced electrons were extracted to the other electrode by circuit to inhibit hole-electron recombination, and thus improve the mineralization efficiency. Owing to the excellent porosity and conductivity, CA could act as very effective adsorbent and electrode materials. Under applied positive potential, negative-charged dye-molecules would be attracted to the positive electrode, enhancing adsorption. The TiO_2_ photocatalyst mineralized the dye, which avoided saturated adsorption of CA material, while CA adsorbed and enriched dye pollutant to keep high pollutant concentration on the TiO_2_ surface to make the photocatalytic reaction proceed rapidly. Photocatalysis and electroadsorption synergistically ensured highly efficient high-concentration dye solution decontamination, which is important not only in academics, but also in industrial engineering.

**Figure 5:**
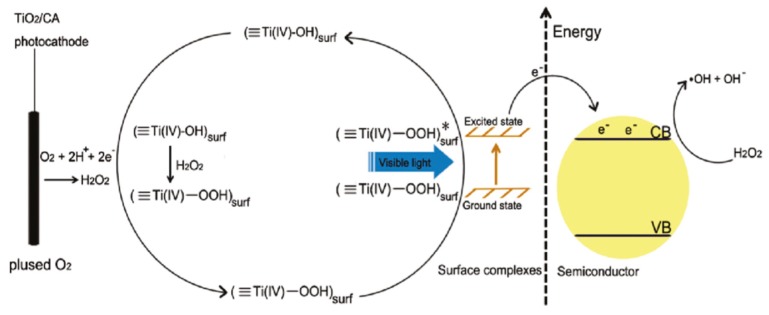
Mechanism of *in-situ* induced visible-light photoelectrocatalysis from molecular oxygen on carbon aerogels-supported TiO_2_. Copied with the permission from [87], Copyrights 2011 ACS [87].

Furthermore, Zhao and co-workers discovered that TiO_2_/CA material could have the capacity to act as an effective photoelectrode to extend the TiO_2_ absorption spectrum to the visible-light region and mineralize Rhodamine 6G dye pollutants under visible-light irradiation in an aerobic condition under −0.9 V bias [87]. They figured out that H_2_O_2_ was generated on the electrode surface by the reduction of dioxygen. Further, the *in-situ* generated H_2_O_2_ coordinated with TiO_2_, forming a visible-light responsive surface Ti–peroxide complex. Upon light irradiation, this surface complex was excited and injected electrons to the TiO_2_ conduction band. The conduction band electrons’ decomposed surface adsorbed H_2_O_2_ to hydroxyl radicals. The hydroxyl radicals that possess strong oxidative ability (E_1/2_ = 2.4 V vs. NHE) can be directly applied to mineralize pollutants. This mechanism was shown in Figure 5. To demonstrate the efficiency of this photoelectrocatalysis system, azo dye Rhodamine 6G was applied as a model pollutant in the activity test. The results showed that, upon –0.9 V bias and visible-light irradiation (λ > 420 nm), the TiO_2_/CA photocathode could reach removal efficiency for Rhodamine 6G at 90.3% and TOC at 83.3% in 300 min. GC-MS (Gas Chromatography- Mass Spectroscopy) and HPLC (High-performance Liquid Chromatography) tracing of intermediates indicated that less intermediates were generated under photoelectrocatalytic conditions than under traditional photocatalytic conditions. This catalyst system demonstrated excellent pollutant removal efficiency and low energy consumption. More importantly, this example provided us a new approach to explore the visible-light responsive unconventional TiO_2_-based photocatalytic materials.

**Figure 6:**
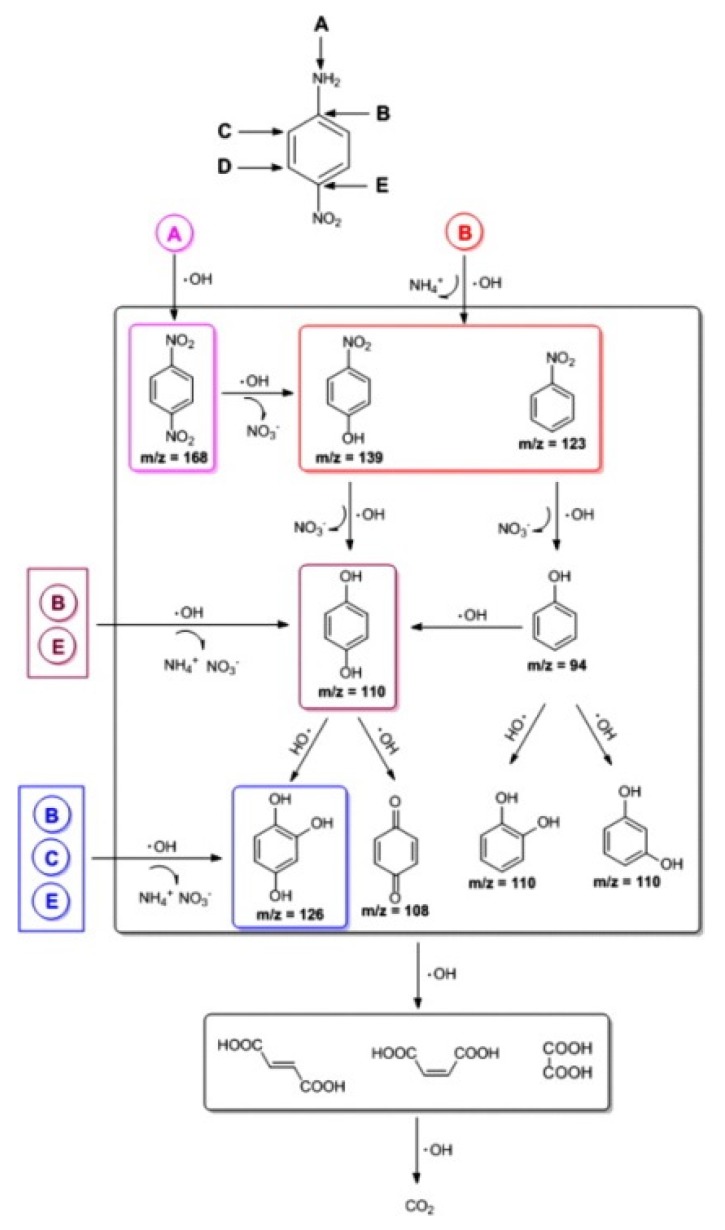
Mechanism of the decomposition of intermediates in TiO_2_ nanorod cluster (NRC)/CA photoelectrocatalysis over p-nitroaniline pollutants. Copied with the permission from [88], Copyrights 2013 Elsevier [88].

Zhao and co-workers reported that TiO_2_ nanorod cluster (NRC) material could be loaded on carbon aerogel by a hydrothermal/calcination method [88]. The as-prepared TiO_2_ NRC/CA composite material could be used as photocathode for the decomposition and mineralization of a notorious pollutant p-nitroaniline. They compared the pollutant removal efficiency and activity of TiO_2_ NRC/CA with TiO_2_ NRC/graphite material. The results indicated that TiO_2_ NRC/CA material could provide superior 98.2% PNA (p-Nitroaniline) removal efficiency in 180 min under visible-light irradiation with −0.6 V bias in comparison with 79.8% for TiO_2_ NRC/graphite. From various characterization experiments, the authors ascribed the higher efficiency of TiO_2_ NRC/CA to the CA’s three-dimensional structure, which provided greater surface area and larger conductivity. More TiO_2_ photocatalyst could be loaded on CA rather than on graphite. Further, more H_2_O_2_ and hydroxyl radicals could be formed on electrode surface. Owing to the greater level of TiO_2_ loading, more surface Ti–peroxide complex and photocurrent could be yielded under visible-light irradiation. All these factors made TiO_2_ NRC/CA excellent photocathode materials for p-nitroaniline pollutants mineralization. Moreover, the authors proposed the decomposition pathways for this photoelectrocatalytic system as shown in Figure 6. They discovered the new important intermediates 1,2,4-trihydroxybenzene during the decomposition processes of p-nitroaniline and reasonably explained its mineralization mechanism.

**Figure 7:**
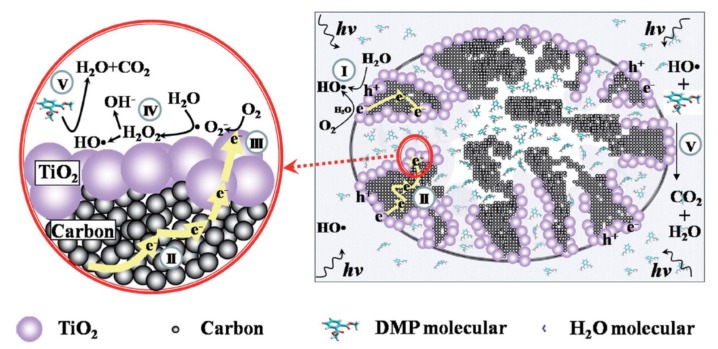
Schematic illustration of dimethyl phthalate (DMP) degradation in TiO_2_/CA-9.3 under light irradiation. The red ring describes the reactions occurring in the secondary pores. The processes include the following: (I) on the external surface, OH• formation with H_2_O reduction and oxidation; (II) the photoelectrons generated on the external surface migrate to the internal surface through the carbon layer; (III) on the internal surface, the photoelectrons are trapped by O_2_ to produce O_2_^•−^; (IV) HOc are generated with the participation of H_2_O and O_2_^•−^ and (V) DMP is oxidized by active HO• on both the external and internal surface. Copied with the permission from [89], Copyrights 2016 RSC [89].

Shi and co-workers discovered that TiO_2_ photocatalytic activity of DMP (dimethyl phthalate) degradation could be enhanced by hybridizing a CA material and constructing secondary hydrophilic pores [89]. They synthesized TiO_2_/CA composite photocatalysts with different CA pore diameters. They discovered that materials comprising CA with 9.3 nm mesopores exhibited the highest photocatalytic degradation efficiency, while CA with smaller pore diameters of 3.4 and 4.3 nm generated inferior results even compared with the pristine TiO_2_ photocatalyst. Contact angle analysis and DMP adsorption tests indicated that CA-9.3 possessed the least ability to adsorb DMP owing to its larger wettability and hydrophilicity. However, as TiO_2_ nanoparticles dispersed in both inner and outer space of CA-9.3, photo-induced electrons on the outer surface migrated to the inner surface through CA conductive porous structure, inhibiting electron-hole recombination and improving the charge separation process. Thus, the generation of hydroxyl radicals by photo-induced holes on the outer surface was enhanced by this effect, which increased the DMP photodegradation rate. Moreover, the generation rate of hydroxyl radical by other routes was also increased. In the inner space of hydrophilic secondary pore, dioxygen, photo-electrons, and water molecules were enriched by this micro-reactor. This encapsulation effect enhanced the hydroxyl radical generation by the dioxygen reduction route in comparison with the lack of porous structure of pristine TiO_2_. This mechanism was illustrated in Figure 7. 

**Figure 8:**
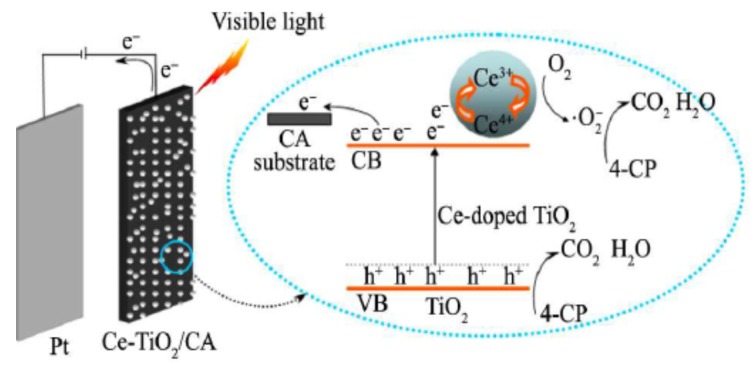
Schematics of the mechanism of Ce-TiO_2_/carbon aerogel electrode in the photoelectrocatalytic degradation of 4-chlorophenol pollutant. Copied with the permission from [90], Copyrights 2018 Elsevier [90].

Wang and co-workers reported that Ce-doped TiO_2_/CA could be applied as potent visible-light responsive electrosorptive photocatalytic materials for 4-chlorophenol degradation [90]. They discovered that cerium doping could introduce defect sites above the top of the valence band red-shifting TiO_2_ absorption spectrum from 387 nm to 532 nm. Furthermore, carbon aerogel was hybridized with Ce-TiO_2_ photocatalyst by an impregnation method. The as-synthesized Ce-TiO_2_/CA material was used as photoelectrode to degrade 4-chlorophenol under 0.6 V bias and 500 W Xenon lamp irradiation. This material exhibited 75 times larger photocurrent than Ce-TiO_2_/FTO (fluorine-doped tin oxide), which was ascribed to the lesser electron-hole recombination rate by the CA porous structure, which facilitated the charge separation processes, as mentioned above. Furthermore, for the 4-chlorophenol (4-CP) degradation, Ce-TiO_2_/CA generated 97.3% 4-CP removal in 4.5 h, in sharp contrast with 65.4% for Ce-TiO_2_/FTO under other identical photoelectrochemical conditions. This enhancing effect was mainly because of the highly-developed porous structure and excellent conductivity, which were pivotal for 4-CP adsorption and the charge separation and migration processes. This mechanism was illustrated in Figure 8.

**Figure 9:**
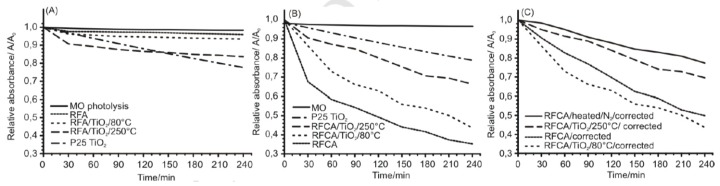
Photocatalytic results of (**A**) resorcinol–formaldehyde aerogel (RFA) and its composite, (**B**) resorcinol–formaldehyde carbon aerogel (RFCA) and its composites, and (**C**) specific surface area and heating corrected data of RFCA and its composites. Copied with the permission from [91], Copyrights 2019 Elsevier [91].

Szilágyi and co-workers reported an atomic-layer-deposition (ALD) method to prepare resorcinol–formaldehyde aerogel (RFA) and resorcinol–formaldehyde carbon aerogel (RFCA)/TiO_2_ composite photocatalysts [91]. They initially prepared RFA and RFCA by the modified Pekala’s three-step method; the first step to synthesize RF hydrogel, the second step dry RF hydrogel in supercritical condition forming RFA, and the last step to yield RFCA by sintering RFA under a N_2_ atmosphere at a high temperature. The as-synthesized RFA and RFCA were utilized as substrates for ALD of TiO_2_ at 80 °C and 250 °C. TiO_2_ prepared at 80 °C was amorphous, while that at 250 °C was crystalline. Furthermore, the authors conducted the photo-degradation experiments of methyl orange dye. Surprisingly, they discovered that RFCA/amorphous TiO_2_ exhibited better photocatalytic activity than RFCA/crystalline TiO_2_. Further, the RFCA material without TiO_2_ deposition exhibited the best photocatalytic activity towards methyl orange degradation. These results were shown in Figure 9. The authors ascribed these unconventional results to the fact that the oxide deposition reduced the surface area and the functional group content of RFCA, thus blocking and decreasing the activity sites. The ALD treatment at a higher temperature more significantly deteriorated the RFCA porous structure and decreased its functional group contents. This report was interesting, demonstrating that amorphous carbonaceous material such as resorcinol–formaldehyde carbon aerogel could not only exhibit semiconductive photocatalytic activity, but also generate higher ability towards organic pollutant degradation under illumination than traditional metal-oxide materials.

The performance of the state-of-the-art examples of TiO_2_/CA photo(electro)catalysis is summarised in Table 1.

Besides the activity improvements of the TiO_2_/CA photo(electro)catalyst, its life cycle assessment (LCA) of the environmental impacts should also be considered. Although we lack the data of the environmental effects of the CA synthesis and the hybrid process of TiO_2_ and CA nano-materials, according to the previously reported LCA of environmental impacts for seven different fabrication routes of TiO_2_ nanomaterials by mass unit, surface-area unit, and photocatalytic activity unit [92], or the reported LCA cradle to gate environmental impacts of five different non-doped and metal-doped TiO_2_ nanomaterials prepared by the sol–gel method by the function unit of photocatalytic carbamazepine and methyl orange degradation [93], we can infer that the TiO_2_/CA photo(electro)catalyst will generate more environmental impacts because the fabrication of CA materials requires an intensive energy-input process and the use of a considerable amount of organic precursor resorcinol and formaldehyde, while the use of formaldehyde will contribute to environmental concern. Further, during the photo(electro)catalytic process of pollutant removal, the added bias would generate much greater consumption of electricity energy. However, the greatly enhanced pollutants removal efficiency would come as a trade-off to the added extra environment impacts for TiO_2_/CA materials compared with non-doped TiO_2_ photocatalysts. Further, a thorough investigation using quantitative software to assess the environmental impacts of the TiO_2_/CA photo(electro)catalyst “from cradle to grave” based on the function unit of the photo(electro)catalytic removal of certain organic pollutants by LCA should be conducted in the future.

## 3. Conclusions

We have outlined the state-of-the-art examples of carbon aerogels materials hybridized with TiO_2_ applied as both catalysts for photoelectrochemical cells and photocatalysts for environmental pollutants degradation. Although still in its blossoming stage in comparison with other traditional inorganic metal oxides, metal chalcogenides, and surface plasmonic metallic photocatalysts and photo-electrocatalysts, CA-modified TiO_2_ material still exhibited miscellaneous advantages. Firstly, carbon aerogels modification could greatly increase the surface area of TiO_2_ owing to the great porosity and highly-developed hierarchical porous structures. The increase of surface area would enhance the adsorption of pollutants on TiO_2_. Furthermore, the introduction of porous structure would enhance electron-hole pair separation by the facilitated TiO_2_ photoelectron transfer between inner surface and outer surface in the interconnected pore structure. Secondly, owing to CA’s excellent conductivity, upon hybridization with CA, TiO_2_ semiconductive material became much more conductive, and the electron-transfer impedance was greatly reduced. This effect was crucial for the increase of photocurrent during the photoelectrochemical degradation of organic pollutants. Last, but not least, the doping of carbon aerogel would introduce defect sites in TiO_2_ bulk solid, thus generating a dopant energy level in the top of the valence band and in the bottom of the conduction band, thus providing the narrower band gap for more efficient photon absorption. This would generate an apparent red-shift phenomenon for TiO_2_, extending its absorption limit from 387 nm to approximately 532 nm. Compared with other crystalline carbonaceous materials, metal oxides, metal chalcogenides, and metal surface plasmonic photocatalysts, CA/TiO_2_ photocatalysts still have a long way to proceed for more mature applications in environment remediation. The main limitations and challenges for CA/TiO_2_ photocatalysts include the following aspects. Initially, the degradation efficiency should be further improved to fulfill the requirements of practical use in industry wastewater treatment. To meet this end, an elaborate, intricate, and novel synthesizing method should be developed. Much higher performance of hydroxyl radical generation and pollutants adsorption should be achieved. Secondly, in order to improve the activity of the composite photocatalyst, the mechanism of how the photoelectrons, photo-induced holes, dioxygen, water, organic pollutants, and the degradation intermediates interacted in the composite photocatalyst, and in particular, how the porous structure influenced the whole photocatalytic process should be more clearly demonstrated. We do believe that if these issues can be resolved successfully, the future of CA/TiO_2_ photocatalysis would be promising.

## Figures and Tables

**Table 1:** Summary of photo(electro)catalytic performance of TiO_2_/carbon aerogel (CA) materials for pollutants removal. NRC, nanorod cluster; RFCA, resorcinol–formaldehyde carbon aerogel.

Catalyst	Condition	Pollutant	Removal Rate Constant	Ref.
TiO_2_/CA	365 nm UV-irradiation under −0.6 V bias	Methylene Blue (150 mg/L)	10.27 × 10^^−3^^ min^−1^	[84]
TiO_2_/CA 125	300 W Hg lamp	Methyl Orange (10 mg/L)	46.2 × 10^−3^ min^−1^	[85]
TiO_2_/CA	80 W UV light (320–400 nm, peak at 365 nm) 0.6 V bias	Alizarin Red (400 mg/L)	9.24 × 10^−3^ min^−1^	[86]
TiO_2_/CA	300 W Xe lamp (420–800 nm) 100 mW/cm^2^ −0.9 V bias	Rhodamine 6G (50 mg/L)	3.61 × 10^−3^ min^−1^	[87]
TiO_2_NRC/CA	500 W Xe lamp (peak at 420 nm) −0.6 V bias	p-Nitroaniline (150 mg/L)	23.1 × 10^−3^ min^−1^	[88]
TiO_2_/CA-9.3	300 W Xe lamp (320–700 nm peak at 420 nm) 85 mW/cm^2^	Dimethyl phthalate (2 mg/L)	12.6 × 10^−3^ min^−1^	[89]
Ce-TiO_2_/CA	500 W Xe lamp 0.6 V bias	4-Chlorophenol (100 mg/L)	9.24 × 10^−3^ min^−1^	[90]
RFCA/TiO_2_/80 °C	2 parallel UV lights (18 W UV-A blacklights)	Methyl Orange (8 × 10^−5^ mol/L)	3.3 × 10^−3^ min^−1^	[91]
